# Refixation proximaler vorderer Kreuzbandläsionen und Augmentation mit „internal brace“: Technische Notiz

**DOI:** 10.1007/s00064-025-00919-4

**Published:** 2025-10-30

**Authors:** Louisa Bell, Christian Egloff

**Affiliations:** 1 Abteilung Kniechirurgie, Schulthess Klinik, Lengghalde 2, 8008 Zürich, Schweiz; 2 Department of Clinical Research, University of Basel, Basel, Switzerland

**Keywords:** Vorderes Kreuzband, Proximale Kreuzbandruptur, ACL-Refixation, Knie, Technische Notiz, Anterior cruciate ligament, Proximal cruciate ligament rupture, ACL repair, Knee, Technical note

## Abstract

**Operationsziel:**

Die Refixation von Läsionen des vorderen Kreuzbandes (VKB) und Augmentation mit Internal Brace® (Arthrex Inc., Naples, FL, USA) hat in den letzten Jahren zunehmend an Bedeutung gewonnen. Das Ziel besteht darin, die native Bandstruktur zu erhalten und das klinische Ergebnis zu verbessern.

**Indikationen:**

Die Indikation stellen proximale VKB-Rupturen vom Sherman-Typ I und II in der Akutphase nach dem Unfallereignis dar. Bei Multiligamentverletzungen kann eine Bandaugmentation zu einer erhöhten Gelenkstabilität beitragen.

**Kontraindikationen:**

Eine VKB-Refixation sollte nicht bei Rupturen des distalen oder mittleren Drittels durchgeführt und posttraumatisch nicht um mehr als 4 Wochen verzögert werden, um eine Einheilung zu gewährleisten.

**Operationstechnik:**

Die Ruptur wird durch ein ultrahochmolekulares Polyethylenband überbrückt und das VKB an den femoralen Footprint readaptiert.

**Weiterbehandlung:**

Die Weiterbehandlung ist funktionell mit einer 2‑ bis 3‑wöchigen Teilbelastung von 15 kg an Unterarmgehstützen. Aktivitäten mit hoher Belastung sollten in den ersten 6 Monaten postoperativ vermieden werden.

**Ergebnisse:**

Die klinischen Ergebnisse der VKB-Augmentation mit Internal Brace® zeigen eine leicht erhöhte Rerupturrate im Vergleich zur VKB-Ersatzplastik bei guten bis hervorragenden funktionellen Ergebnissen.

## Abstract (en)

**Objective:**

Refixation of anterior cruciate ligament (ACL) tears and augmentation using an Internal Brace® (Arthrex Inc., Naples, FL, USA) has become increasingly popular in recent years. The aim is to preserve the native ligament structure and, thus, improve clinical outcomes.

**Indications:**

Indications are proximal ACL tears of Sherman type I and II in the acute phase postinjury. For multiligament injuries, internal brace augmentation can contribute to increased joint stability.

**Contraindications:**

ACL refixation is not recommended for distal or middle segment tears and should not be delayed for more than 4 weeks posttrauma to ensure adequate healing.

**Surgical technique:**

The rupture is bridged by an ultrahigh-molecular-weight polyethylene tape to reattach the ACL to the femoral footprint.

**Postoperative management:**

Functional postoperative treatment involves partial weight-bearing of 15 kg on crutches within 3 weeks postoperatively. High-impact activities should be avoided for the first 6 months postoperatively.

**Results:**

The clinical results of ACL augmentation with an Internal Brace® show a slightly increased re-rupture rate compared to ACL replacement, with good to excellent functional outcomes.

## Vorbemerkungen

Die Ruptur des vorderen Kreuzbandes (VKB) zählt zu den häufigsten Verletzungen des Kniegelenks mit einer Inzidenz von 85 Fällen pro 100.000 Personen im Alter von 16 bis 39 Jahren [5]. Die VKB-Ersatzplastik stellt das derzeitige operative Standardverfahren dar, weist jedoch Nachteile wie den Verlust der Bandpropriozeption sowie mögliche Komplikationen an der Sehnenentnahmestelle auf [1, 7, 13]. Die primäre VKB-Refixation mit Erhalt der nativen Bandstruktur hat daher in den letzten Jahren zunehmend an Bedeutung gewonnen [27]. Im Jahr 1895 berichteten Mayo-Robson et al. [21] erstmals über gute postoperative Ergebnisse nach offener VKB-Refixation; es wurden jedoch hohe Versagensraten festgestellt [13]. Eine Herausforderung ist die Heilung des VKB in der intraartikulären Synovialflüssigkeit, welche die Remodellierung und Bildung einer stabilen Kollagenbrücke erschwert [20]. Die Prognose richtet sich nach der Morphologie der Ruptur, wobei die Indikation für eine Refixation auf proximale Rupturen mit einem distalen Bandanteil von mindestens 75–90 % (Sherman-Klassifikation Typ 1 bis 2) beschränkt ist [14, 22]. Es sind in den letzten Jahren verschiedene Refixationstechniken entwickelt worden, darunter die Augmentation mit Internal Brace® (Arthrex Inc., Naples, FL, USA), die Ankerrefixation, die dynamische intraligamentäre Stabilisierung (Ligamys®, Mathys AG, Bettlach, Schweiz) und die Überbrückung des rupturierten Bandes mit Biomaterial (Bridged Enhanced ACL Repair [BEAR]) [2, 20]. Das Internal Brace® stellt eine häufig verwendete Verstärkung des rupturierten VKB dar, bei der die rupturierten Bandanteile femoral reinseriert werden und durch ein ultrahochmolekulares Polyethylenband als sekundärer Bandstabilisator verstärkt wird. Im Vergleich zum Standardverfahren der VKB-Ersatzplastik sind ein leicht erhöhtes Rerupturrisiko [2, 25, 27], jedoch eine kürzere Operations- und Rehabilitationszeit, geringe Komplikationsraten und eine verbesserte frühpostoperative Kniefunktion beschrieben [3, 4, 15, 19, 25].

## Operationsprinzip und -ziel

Die VKB-Augmentation mit Internal Brace® ist eine arthroskopisch gestützte Technik zur Refixation proximaler VKB-Rupturen, bei der die Diskontinuität durch einen geflochtenen Polyethylenfaden (z. B. FiberTape®, Arthrex Inc., Naples, FL, USA, oder Ethibond®, Ethicon, Somerville, NJ, USA) überbrückt wird. Die Ziele sind eine Steigerung der mechanischen Bandstabilität, Wiederherstellung der physiologischen Kinematik sowie eine beschleunigte postoperative Rehabilitation.

## Vorteile

- Das Internal Brace® bietet einen mechanischen Schutz während der Remodellierungsphase und übernimmt danach die Funktion als sekundärer Bandstabilisator. Durch einen Erhalt der Blutversorgung werden die Revaskularisierung und folglich Einheilung gefördert.
- Für die VKB-Augmentation mittels Internal Brace® ist keine Sehnenentnahme erforderlich, wodurch eine Morbidität an der Sehnenentnahmestelle und muskuläre Schwäche reduziert werden.
- Die Bandaugmentation ermöglicht einen Erhalt des nativen Gewebes einschließlich der propriozeptiven Bandeigenschaften [1, 7, 27]. Dies kann zu einer beschleunigten Rehabilitation und erleichterten Frühmobilisation beitragen [4, 13].
- Durch die interne Abstützung wird eine erhöhte Zugfestigkeit generiert, wodurch biomechanisch höhere Spitzenbelastungen toleriert werden [18].
- Eventuelle Revisionsoperationen werden als weniger kompliziert eingeschätzt, und eine spätere VKB-Ersatzplastik ist weiterhin möglich [10].
- In der Situation von Multiligamentverletzungen bietet die Bandaugmentation die Möglichkeit der minimalinvasiven Therapie, um das Risiko postoperativer Komplikationen wie der Arthrofibrose zu verringern [8].

## Nachteile

- Im Vergleich zur VKB-Ersatzplastik ist eine leicht höhere Wahrscheinlichkeit des Transplantatversagens beschrieben mit einer Rerupturrate von durchschnittlich 10,4 % [27].
- Der Operationszeitpunkt ist zeitlich auf bis zu 4 Wochen nach dem Unfallereignis limitiert, da das Einheilungspotenzial nur in der Akutphase gewährleistet ist.
- Die Durchführbarkeit ist von der Rissmorphologie abhängig und nicht bei insuffizienter Gewebequalität oder distalen Rupturen angeraten [16, 22].

## Indikationen

- Proximale Rupturen des VKB weisen ein verbessertes Einheilungspotenzial auf, wodurch nach der Klassifikation von Sherman bei Rupturen des Typs I (d. h. proximale Avulsion mit einem Bandrest von 90 %) und II (d. h. Ruptur im oberen Banddrittel mit distalem Bandrest von mindestens 75 %) der Erhalt des VKB möglich ist [6, 16, 22].
- Das Internal Brace® kann bei akuten Rupturen, in einem Zeitfenster innerhalb der ersten 4 Wochen nach der Verletzung, angewendet werden. In diesem Zeitraum ist das Einheilungspotenzial gegeben und ein VKB-Erhalt möglich.
- Bei der Indikationsstellung sollten die Gewebequalität, der Verletzungsmechanismus und das Patientenalter berücksichtigt werden. Wenngleich es für Erwachsene keine Altersbeschränkung gibt, wurde bei Patienten in einem Alter von > 21 Jahren eine geringere Versagens- und Komplikationsrate beschrieben [26].
- Möglicher Einsatz der Augmentation mit Internal Brace® bei multiligamentären Kniegelenkverletzungen zur Steigerung der Stabilitätsverhältnisse [8].

## Kontraindikationen

- Die Augmentation mit Internal Brace® ist bei Mittelsubstanz- und distalen Rissen kontraindiziert, da hier keine stabile Refixation erzielt werden kann.
- Eine relative Kontraindikation sind VKB-Rupturen, bei denen das Unfallereignis bereits mehr als 4 Wochen zurückliegt.
- Bei Patienten mit noch nicht abgeschlossenem Skelettwachstum und offenen Wachstumsfugen liegen bislang keine ausreichenden Daten vor. Erste Studien deuten auf die Möglichkeit einer vorübergehenden Augmentation mit Entfernung des Internal Brace® 3 Monate postoperativ hin [23].

## Patientenaufklärung

- Allgemeine Operationsrisiken einschließlich des Risikos von Infektionen, Nerven- oder Weichteilschäden, ossären Verletzungen und Sensibilitätsstörungen.
- Risiko der Reruptur sowie postoperativen Arthrofibrose.
- Verwendung von Fremdmaterial mit Implantation eines metallischen TightRope®-Buttons (Arthrex Inc., Naples, FL, USA) zur transossären Fixierung.
- Intraoperative Entscheidung über den Umfang des Eingriffs bei zusätzlichen Meniskusläsionen oder über die Alternative, einen Kreuzbandersatz durchzuführen, sofern eine Bandrekonstruktion aufgrund der Rissmorphologie nicht möglich ist.
- Nachbehandlungsschema mit Limitation der postoperativen Belastbarkeit (s. Postoperative Behandlung).

## Operationsvorbereitungen

- Native Magnetresonanztomographie(MRT)-Diagnostik sowie Röntgenaufnahmen (a.-p., lateral, ggf. Patella-Defilee) des Kniegelenks.
- Rasur des Beins auf der zu operierenden Seite.
- Antibiotikaprophylaxe als gewichtsadaptierte Einzeldosis nach klinikinternem Standard.
- Oberschenkelblutsperre mit möglichst proximaler Anlage.

## Instrumentarium

- Arthroskopieturm mit entsprechendem arthroskopischen Instrumentenset (inklusive VKB-Zielinstrumentensystem und Scorpion™-Fadeneinzugshilfe).
- Zur besseren Instrumentierbarkeit durch das Arbeitsportal kann optional eine PassPort Button™-Kanüle, 8 mm × 2 cm eingesetzt werden.
- Zur Augmentation des rupturierten VKB wird ein ACL-Repair TightRope® mit FiberTape® genutzt, welches femoral mittels eines TightRope®-Buttons fixiert wird.
- Das proximale VKB wird durch eine Schlaufennaht angeschlungen. Hierfür wird ein FiberRing™ mit einer Shuttleschlaufe angewendet.
- Zur femoralen Ausleitung des ACL-Repair TightRope®-Systems ist ein 1,3 mm SutureTape®-Faden mit geschlossener Schlaufe erforderlich.
- Zur tibialen Fixierung der VKB-Augmentation kann ein metallischer Button oder eine Interferenzschraube genutzt werden.

Das gelistete Instrumentarium bezieht sich auf das Internal Brace®-System von Arthrex Inc. (Naples, FL, USA).

## Anästhesie und Lagerung

- Die Operation kann sowohl in Allgemein- oder Regionalanästhesie durchgeführt werden. Periphere Nervenleitungsanästhesien zur postoperativen Analgesie sind in der Regel nicht erforderlich [24].
- Die Lagerung erfolgt in liegender Patientenposition mit einer Seitenstütze und optional mit einem elektromotorisch betriebenen Knielagerungsaggregat (Getinge, Rastatt, DE).
- Die sterile Abdeckung ist analog zu der VKB-Ersatzplastik.

## Operationstechnik

**Abb. 1:**
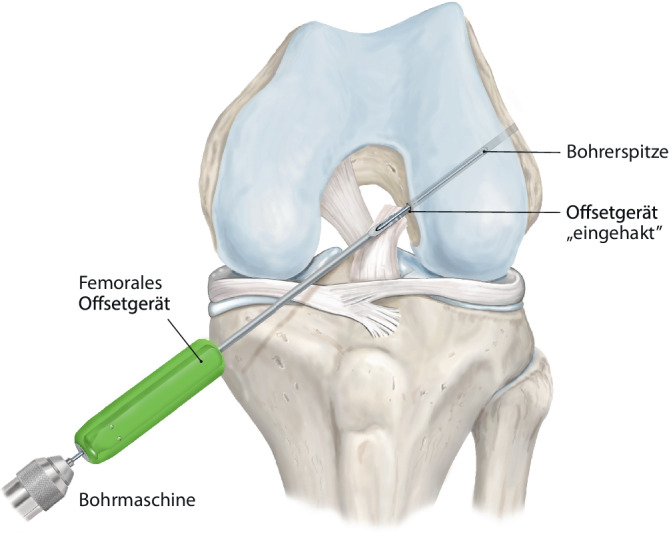
Zunächst erfolgt eine diagnostische Kniearthroskopie über ein hohes anterolaterales Kameraportal und anteromediales Arbeitsportal. Eine Fotodokumentation wird vorgenommen, und meniskale oder chondrale Begleitläsionen werden versorgt. Hiernach wird die VKB-Rissmorphologie beurteilt und auf Basis der Gewebestruktur und Läsionshöhe der Entscheid getroffen, ob eine Reinsertion möglich ist (Abb. 9a). Das proximal rupturierte Bündel und der femorale Footprint werden leicht mit dem Shaver debridiert. Der femorale Offset-Guide wird über das mediale Portal am nativen femoralen Insertionspunkt in 130° Flexion angesetzt. Ein 4 mm Flügelbohrer wird im Bereich des femoralen Footprints angesetzt und ein 4er-Bohrdraht eingebracht

**Abb. 2:**
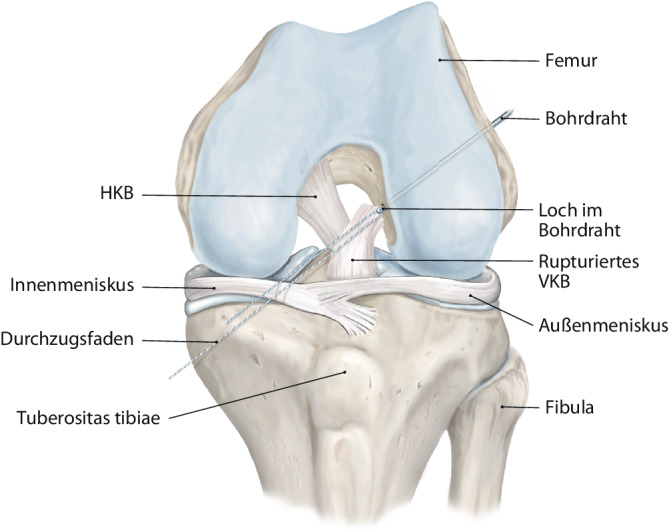
Nach dem Aufbohren des femoralen Kanals mit dem 4er-Bohrdraht wird ein Durchzugsfaden eingezogen

**Abb. 3:**
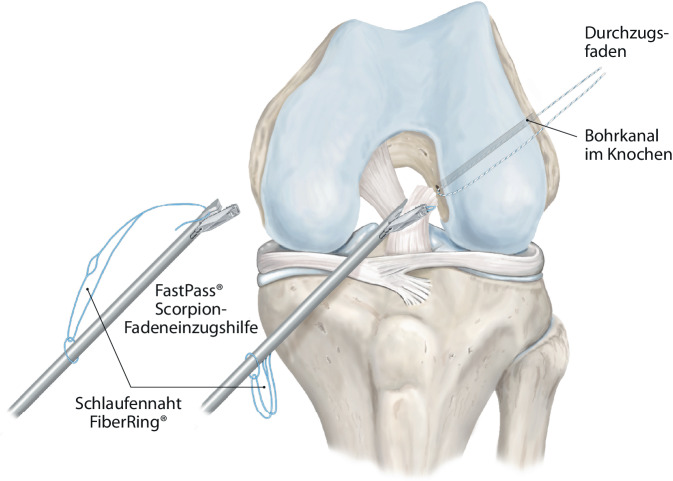
Der VKB-Stumpf wird anschließend mittels FastPass Scorpion™-Fadeneinzugshilfe angeschlungen (vgl. Abb. 9b) und eine Schlaufennaht (FiberRing™-Faden, Arthrex Inc., Naples, FL, USA) befestigt (vgl. Abb. 9c). Es sollten möglichst alle Anteile des VKB-Stumpfes umfasst werden. Zur vereinfachten Handhabung kann eine PassPort™-Kanüle in das anteromediale Arbeitsportal eingebracht werden

**Abb. 4:**
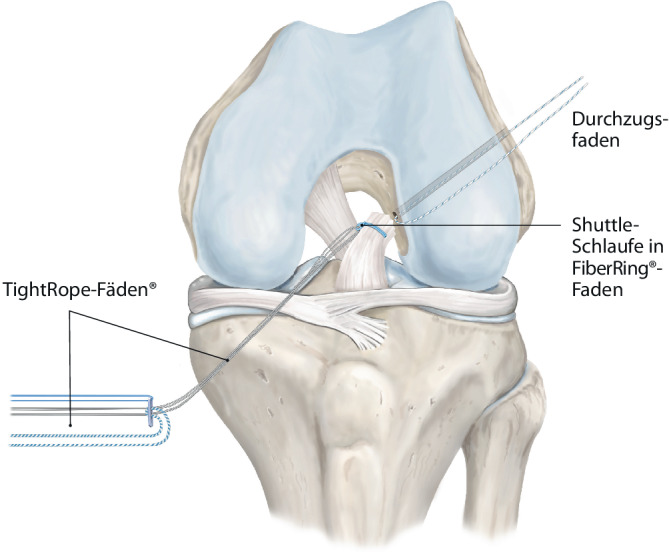
Über eine Shuttleschlaufe in dem FiberRing™-Faden werden die TightRope®-Fäden (Arthrex Inc., Naples, FL, USA) eingezogen

**Abb. 5:**
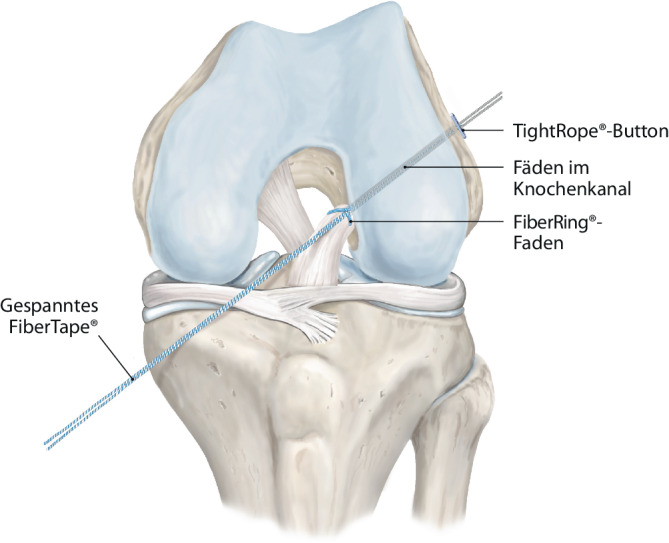
Per Flaschenzugmechanismus mit retrogradem Anspannen wird das FiberTape® (Arthrex Inc., Naples, FL, USA) mit den Armierungsfäden eingezogen, wodurch das VKB an den femoralen Insertionspunkt readaptiert wird. Der TightRope®-Button (Arthrex Inc., Naples, FL, USA) wird transfemoral ausgeleitet und proximal durch Flippen an die Gegenkortikalis angelegt. Die Spannung des VKB-Stumpfes wird mit dem Tasthaken geprüft

**Abb. 6:**
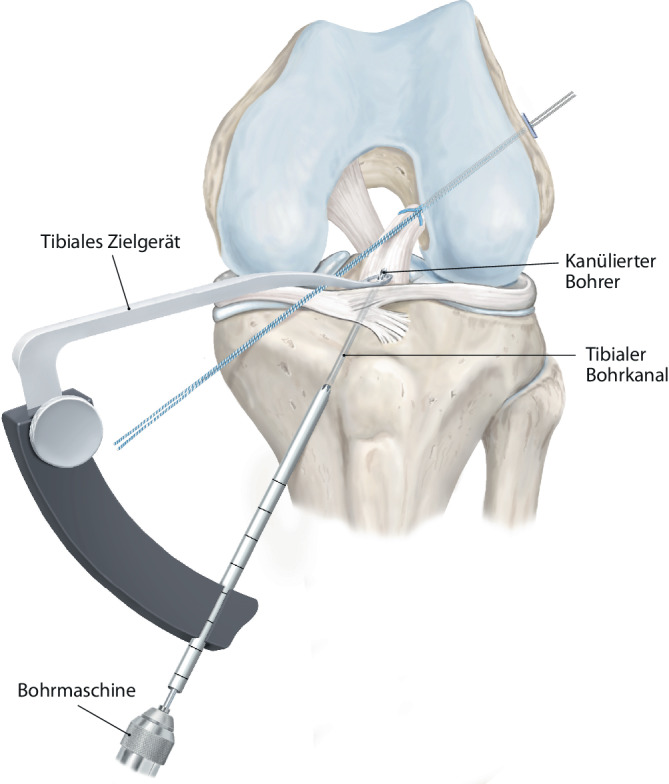
Ein konventionelles tibiales Zielgerät wird zentral im VKB-Stumpf eingebracht, und mit einem 2,4 mm kanülierten Bohrer wird über eine entsprechende Bohrhülse aufgebohrt (vgl. Abb. 9d)

(Abb. 1, 2, 3, 4, 5, 6, 7 und 8).

**Abb. 7:**
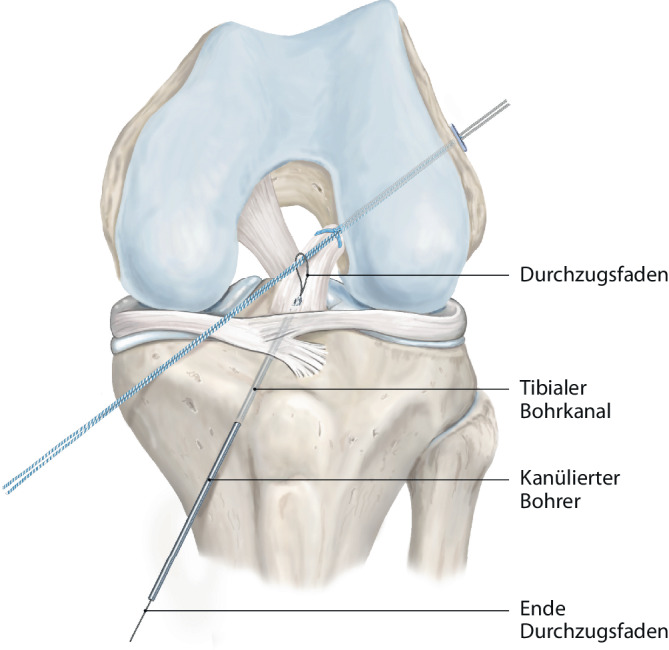
Durch den tibialen Kanal wird ein Zugfaden eingebracht und das FiberTape™ (Arthrex Inc., Naples, FL, USA) angeschlungen

**Abb. 8:**
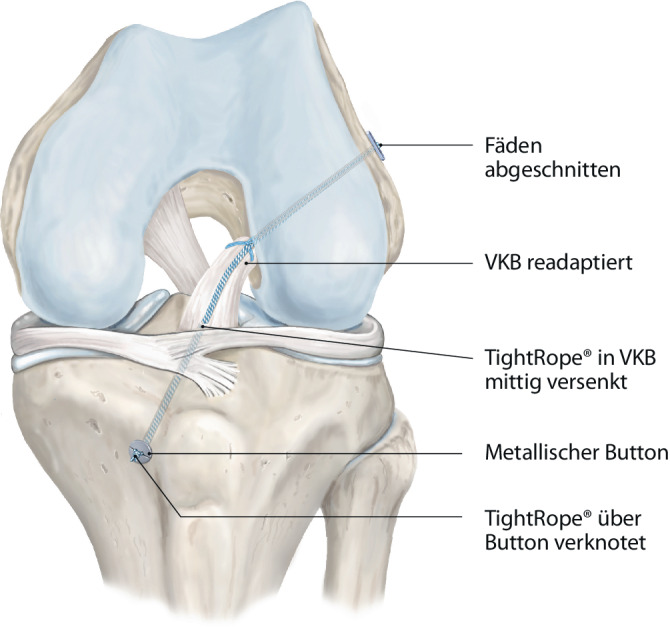
Das FiberTape™ (Arthrex Inc., Naples, FL, USA) wird transtibial nach distal ausgeleitet und über eine Interferenzschraube oder einen metallischen Button in voller Extension fixiert. Intraoperativ erfolgt ein Lachman-Test zur Prüfung der Bandstabilität

**Abb. 9:**
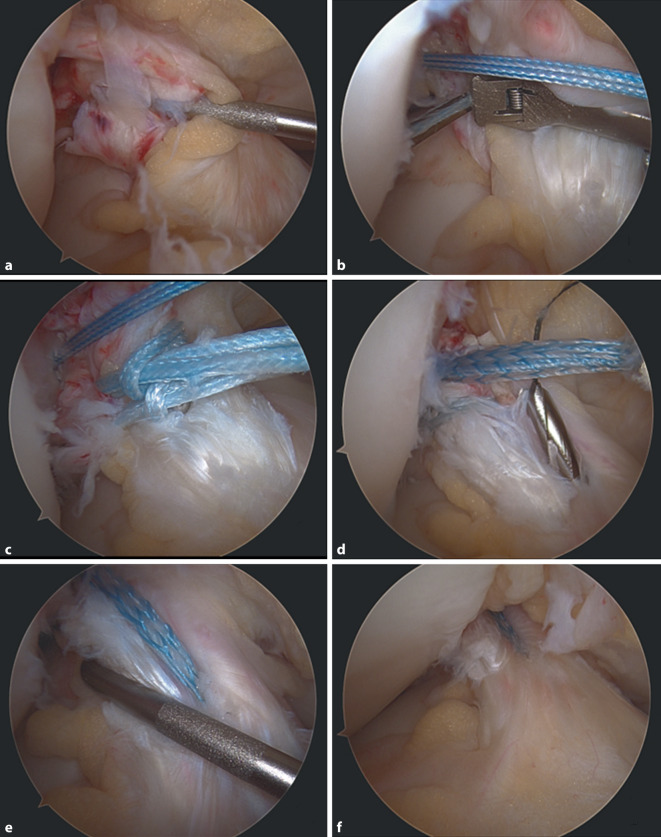
**a**–**f** Intraoperative arthroskopische Dokumentation. **a** Beurteilung der Rissmorphologie des vorderen Kreuzbandes (VKB), **b** Anschlingen des VKB mittels Scorpion™-Fadeneinzugshilfe, **c** Anlage einer doppelten Schlaufennaht, **d** Einbringen eines kanülierten Bohrers tibial, **e** Beurteilung der Bandstabilität in Knieflexion und **f** Knieextension

## Postoperative Behandlung

Postoperativ erfolgt die funktionelle Nachbehandlung mit einer 2‑ bis 3‑wöchigen Teilbelastung von 15 kg an 2 Unterarmgehstützen; hiernach erfolgt eine Vollbelastung. Im Falle einer zusätzlichen Meniskusnaht erfolgt eine Teilbelastung über einen entsprechend längeren Zeitraum von 4 bis 6 Wochen postoperativ. Eine Knieextensionsschiene wird für 5 Tage postoperativ angelegt, um unwillkürliche, abrupte Bewegungen zu vermeiden. Die weitere Nachbehandlung erfolgt ohne Orthese mit freiem Bewegungsumfang. Für die Dauer der Gehstocknutzung wird eine Thromboembolieprophylaxe angeraten. Eine konventionell-radiologische Kontrolle sollte intraoperativ oder unmittelbar postoperativ erfolgen, um die korrekte kortikale Lage des femoralen Buttons zu dokumentieren. Zur optimierten Linderung perioperativer, entzündlicher Prozesse kann eine Vitamin-C-Supplementierung in Form von Redoxon® (Bayer Pharma AG, Leverkusen, DE) genutzt werden. Durch die Unterstützung einer Physiotherapie wird die Schwellung in den ersten 2 Wochen reduziert und die vollständige Extension erlangt. Ein progressiver Kraftaufbau erfolgt ab der sechsten Woche postoperativ, wobei Aktivitäten mit hoher Stoßbelastung für 4 bis 6 Monate postoperativ vermieden werden sollten. Die Entscheidung über den Zeitpunkt der Rückkehr zum Sport erfolgt in Abhängigkeit von funktionellen und isokinetischen Kraftmessungen.

## Fehler, Gefahren, Komplikationen

- Reruptur der VKB-Refixation: Ein konservativer Behandlungsansatz kann zunächst versucht werden, sofern eine suffiziente klinische Kniestabilität besteht. Andernfalls ist eine sekundäre VKB-Rekonstruktion mit autologem Sehnentransplantat möglich [11].
- Arthrofibrose: Die Verbesserung des Bewegungsumfangs kann durch eine geschlossene oder arthroskopische Arthrolyse erzielt werden.
- Folgeverletzungen: Diese können in Abhängigkeit der Binnenläsion adressiert werden. Im Falle einer nachträglichen meniskalen Läsion kann eine Teilmenisektomie oder Nahtversorgung erfolgen.

## Ergebnisse

Der Einsatz eines Internal Brace® zur Augmentation akuter, proximaler VKB-Rupturen wird durch die Ergebnisse in der Literatur gestützt [3, 4, 6, 9, 10, 12, 15, 19, 25, 27]. Im Vergleich zur VKB-Plastik wurden vergleichbare oder bessere frühfunktionelle Ergebnisse beschrieben, verbunden mit einer leicht erhöhten Versagensrate [9, 25–27]. Braithwaite et al. [2] zeigten in einer aktuellen Metaanalyse, in die 18 Studien zur primären VKB-Refixation mit einer Mindestnachbeobachtungszeit von 2 Jahren einbezogen wurden, gute bis hervorragende Ergebnisse mit einem durchschnittlichen gewichteten International Knee Documentation Committee(IKDC)- und Lysholm-Score von 91,7 (95 %-KI: 89,6–93,8) bzw. 94,7 (95 %-KI: 92,7–96,7). Van der List et al. [17] stellten in einer Metaanalyse die verschiedenen Techniken zur Refixation des VKB gegenüber und zeigten durchweg gute funktionelle Ergebnisse von > 85 % der maximal erreichbaren Funktionsfähigkeit (einschließlich IKDC, Tegner-Score, Lysholm-Score, Sports subscale oft he Knee Injury and Osteoarthritis Outcome Score [KOOS] und Single Assessment Numeric Score [SANE] zur Kniefunktion). In einer Metaanalyse von 7 randomisierten kontrollierten Studien konnte zudem kein Unterschied zwischen einer arthroskopischen Refixation und einer VKB-Ersatzplastik in Bezug auf KT-1000-Messungen, Bewegungsumfang, funktionelle Ergebnisse und Reoperationsrate nachgewiesen werden [12]. Frühpostoperativ nach VKB-Refixation wurden zudem ein geringeres Schmerzniveau mit einem geringeren Opioidverbrauch, eine schnellere Mobilitätssteigerung und beschleunigte Rehabilitation beschrieben [24]. Die postoperative Regeneration ist gemäß der Beschreibung von Sherman et al. [22] hierbei abhängig von der Rissmorphologie und dem Patienten- bzw. Aktivitätsprofil. Die Forschungsgruppe untersuchte 50 Patienten im Alter von 23 Jahren (Spanne: 15–56), darunter 76 % unter 30 Jahren, die 7 Tage (Spanne: 1–18) posttraumatisch mit einer primären VKB-Refixation versorgt wurden. Auf der Grundlage der postoperativen Ergebnisse wurden 4 Rupturarten unterschieden, wobei eine Ruptur der mittleren Substanz vom Typ IV mit einer schlechteren Prognose verbunden war. Als Risikofaktoren für eine eingeschränkte Erfolgswahrscheinlichkeit wurden zudem fußballbedingte Verletzungen, ein jüngeres Patientenalter und ein ausgeprägter perioperativer Pivot-Shift als Zeichen für eine Rotationsinstabilität identifiziert. In der Literatur wird die Versagensrate der VKB-Refixation derzeit auf durchschnittlich 10,4 % geschätzt bei einer Nachbeobachtungszeit von 2,7 Jahren [27]. Vermeijden et al. zeigten in einem systematischen Review und einer Metaanalyse eine Versagensrate von durchschnittlich 8 %, wobei diese in der jüngeren Altersgruppe höher lag (17 %) als in der älteren (6 %) [25]. Hopper et al. [10] zeigten ein erhöhtes Risiko für eine Reruptur bei einem höheren Marx-Aktivitätslevel vor dem Unfallereignis mit zufriedenstellenden Fünfjahresergebnissen bei 82,4 % der analysierten Patienten. Im eigenen Patientenkollektiv konnten 2 Jahre nach der Operation vergleichbar gute bis hervorragende Ergebnisse beobachtet werden mit einem Trend zu physiologischeren Kniemomenten und einer verbesserten Muskelfunktion in der Ganganalyse [3, 4, 19]. Im Vergleich zur VKB-Ersatzplastik zeigten sich keine Unterschiede bezüglich der isokinetischen Kraft und des funktionellen Leistungsniveaus [3, 4].

Zusammenfassend weist die Augmentation proximaler VKB-Rupturen mit Internal Brace® klinisch Erfolg versprechende Ergebnisse auf. Die intraligamentäre Stabilisierung trägt durch den Erhalt der nativen Bandstruktur und Propriozeption zu verbesserten frühfunktionellen Ergebnissen bei. Zukünftige Forschung sollte ermitteln, inwieweit die VKB-Refixation in Bezug auf Standfestigkeit und Einsatzmöglichkeiten bei jüngeren Patienten weiter optimiert werden kann.

## Data Availability

Die in dieser Studie erhobenen Datensätze können auf begründete Anfrage beim Korrespondenzautor angefordert werden.

## References

[1] Adachi N, Ochi M, Uchio Y et al (2002) Mechanoreceptors in the anterior cruciate ligament contribute to the joint position sense. Acta Orthop Scand 73:330–334. 10.1080/000164702320155356 12143983 10.1080/000164702320155356

[2] Braithwaite C, Hafen TJ, Dean R et al (2024) Outcomes of Primary Anterior Cruciate Ligament (ACL) Repair for Proximal Tears: A Systematic Review and Meta-Analysis. Cureus 16:e59124. 10.7759/cureus.59124 38803739 10.7759/cureus.59124 PMC11129541

[3] Bühl L, Müller S, Nüesch C et al (2023) Ambulatory knee biomechanics and muscle activity 2 years after ACL surgery: InternalBrace(TM)-augmented ACL repair versus ACL reconstruction versus healthy controls. BMC Musculoskelet Disord 24:785. 10.1186/s12891-023-06916-7 37794432 10.1186/s12891-023-06916-7 PMC10548591

[4] Bühl L, Müller S, Nüesch C et al (2023) Functional leg performance 2 years after ACL surgery: a comparison between InternalBrace^TM^-augmented repair versus reconstruction versus healthy controls. J Orthop Traumatol 24:52. 10.1186/s10195-023-00723-5 37735271 10.1186/s10195-023-00723-5 PMC10513977

[5] Diermeier T, Rothrauff BB, Engebretsen L et al (2020) Treatment after anterior cruciate ligament injury: Panther Symposium ACL Treatment Consensus Group. Knee Surg Sports Traumatol Arthrosc 28:2390–2402. 10.1007/s00167-020-06012-6 32388664 10.1007/s00167-020-06012-6 PMC7524809

[6] van Eck CF, Limpisvasti O, ElAttrache NS (2018) Is There a Role for Internal Bracing and Repair of the Anterior Cruciate Ligament? A Systematic Literature Review. Am J Sports Med 46:2291–2298. 10.1177/0363546517717956 28783472 10.1177/0363546517717956

[7] Gokeler A, Benjaminse A, Hewett TE et al (2012) Proprioceptive deficits after ACL injury: are they clinically relevant? Br J Sports Med 46:180–192. 10.1136/bjsm.2010.082578 21511738 10.1136/bjsm.2010.082578

[8] Heitmann M, Akoto R, Krause M et al (2019) Management of acute knee dislocations: anatomic repair and ligament bracing as a new treatment option-results of a multicentre study. Knee Surg Sports Traumatol Arthrosc 27:2710–2718. 10.1007/s00167-018-5317-4 30631909 10.1007/s00167-018-5317-4

[9] Heusdens CHW, Blockhuys K, Roelant E et al (2021) Suture tape augmentation ACL repair, stable knee, and favorable PROMs, but a re-rupture rate of 11 % within 2 years. Knee Surg Sports Traumatol Arthrosc 29:3706–3714. 10.1007/s00167-020-06399-2 33386882 10.1007/s00167-020-06399-2

[10] Hopper GP, Aithie JMS, Jenkins JM et al (2022) Satisfactory patient-reported outcomes at 5 years following primary repair with suture tape augmentation for proximal anterior cruciate ligament tears. Knee Surg Sports Traumatol Arthrosc 30:253–259. 10.1007/s00167-021-06485-z 33582828 10.1007/s00167-021-06485-z PMC8800885

[11] Hopper GP, Wilson WT, O’Donnell L et al (2022) Comparable rates of secondary surgery between anterior cruciate ligament repair with suture tape augmentation and anterior cruciate ligament reconstruction. J exp orthop 9:115. 10.1186/s40634-022-00549-w 36459283 10.1186/s40634-022-00549-w PMC9718902

[12] Li Z (2022) Efficacy of Repair for ACL Injury: A Meta-analysis of Randomized Controlled Trials. Int J Sports Med 43:1071–1083. 10.1055/a-1755-4925 35100655 10.1055/a-1755-4925 PMC9713465

[13] van der List JP, DiFelice GS (2016) Preservation of the Anterior Cruciate Ligament: A Treatment Algorithm Based on Tear Location and Tissue Quality. Am J Orthop 45:E393–E405 28005092

[14] van der List JP, DiFelice GS (2017) Primary repair of the anterior cruciate ligament: A paradigm shift. Surgeon 15:161–168. 10.1016/j.surge.2016.09.006 27720666 10.1016/j.surge.2016.09.006

[15] van der List JP, DiFelice GS (2017) Range of motion and complications following primary repair versus reconstruction of the anterior cruciate ligament. Knee 24:798–807. 10.1016/j.knee.2017.04.007 28549818 10.1016/j.knee.2017.04.007

[16] van der List JP, DiFelice GS (2017) Role of tear location on outcomes of open primary repair of the anterior cruciate ligament: A systematic review of historical studies. Knee 24:898–908. 10.1016/j.knee.2017.05.009 28803759 10.1016/j.knee.2017.05.009

[17] van der List JP, Vermeijden HD, Sierevelt IN et al (2020) Arthroscopic primary repair of proximal anterior cruciate ligament tears seems safe but higher level of evidence is needed: a systematic review and meta-analysis of recent literature. Knee Surg Sports Traumatol Arthrosc 28:1946–1957. 10.1007/s00167-019-05697-8 31486914 10.1007/s00167-019-05697-8 PMC7253375

[18] Massey P, Parker D, McClary K et al (2020) Biomechanical comparison of anterior cruciate ligament repair with internal brace augmentation versus anterior cruciate ligament repair without augmentation. Clin Biomech 77:105065. 10.1016/j.clinbiomech.2020.105065 10.1016/j.clinbiomech.2020.105065 32504897

[19] Müller S, Bühl L, Nüesch C et al (2024) Favorable Patient-Reported, Clinical, and Functional Outcomes 2 Years After ACL Repair and InternalBrace Augmentation Compared With ACL Reconstruction and Healthy Controls: Response. Am J Sports Med 52:NP16–NP18. 10.1177/03635465241247723 38946457 10.1177/03635465241247723

[20] Murray MM, Fleming BC (2013) Biology of anterior cruciate ligament injury and repair: Kappa delta ann doner vaughn award paper 2013. J Orthop Res 31:1501–1506. 10.1002/jor.22420 23818453 10.1002/jor.22420 PMC3750083

[21] Robson AW (1903) VI. Ruptured Crucial Ligaments and their Repair by Operation. Ann Surg 37:716–718 17861289 PMC1431029

[22] Sherman MF, Lieber L, Bonamo JR et al (1991) The long-term followup of primary anterior cruciate ligament repair. Defining a rationale for augmentation. Am J Sports Med 19:243–255. 10.1177/036354659101900307 1867333 10.1177/036354659101900307

[23] Smith JO, Yasen SK, Palmer HC et al (2016) Paediatric ACL repair reinforced with temporary internal bracing. Knee Surg Sports Traumatol Arthrosc 24:1845–1851. 10.1007/s00167-016-4150-x 27141865 10.1007/s00167-016-4150-x

[24] Vermeijden HD, Holuba K, Yang XA et al (2023) Prospective Comparison of Postoperative Pain and Opioid Consumption Between Primary Repair and Reconstruction of the Anterior Cruciate Ligament. Orthop J Sports Med 11:23259671231187442. 10.1177/23259671231187442 37786478 10.1177/23259671231187442 PMC10541769

[25] Vermeijden HD, van der List JP, Benner JL et al (2022) Primary repair with suture augmentation for proximal anterior cruciate ligament tears: A systematic review with meta-analysis. Knee 38:19–29. 10.1016/j.knee.2022.07.001 35870397 10.1016/j.knee.2022.07.001

[26] Vermeijden HD, Yang XA, van der List JP, DiFelice GS (2021) Role of Age on Success of Arthroscopic Primary Repair of Proximal Anterior Cruciate Ligament Tears. Arthroscopy 37:1194–1201. 10.1016/j.arthro.2020.11.024 33220465 10.1016/j.arthro.2020.11.024

[27] Wilson WT, Hopper GP, Banger MS et al (2022) Anterior cruciate ligament repair with internal brace augmentation: A systematic review. Knee 35:192–200. 10.1016/j.knee.2022.03.009 35366618 10.1016/j.knee.2022.03.009

